# Hepatitis A Vaccination Rates and Related Factors in a 2005 Population-based Study in Nonsan, Korea

**DOI:** 10.4178/epih/e2009003

**Published:** 2009-10-12

**Authors:** Eun Young Kim, Baeg Ju Na, Moo Sik Lee, Keon Yeop Kim, Moran Ki

**Affiliations:** 1 Department of Preventive Medicine, School of Medicine, Konyang University, Daejeon, Korea.; 2 Department of Preventive Medicine, School of Medicine, Kyungpook National University, Daegu, Korea.; 3 Department of Preventive Medicine, Eulji University School of Medicine, Daejeon, Korea.

**Keywords:** Hepatitis A, Immunization, Infant, Risk factors, Vaccination

## Abstract

**OBJECTIVES:**

The incidence of clinical hepatitis A has increased in young Korean adults since the mid-1990s. Although hepatitis A vaccinations have been administered in private clinics over the past 10 yr, no data exist on the vaccination rate and relating factors.

**METHODS:**

In 2005, a population-based survey of 12-35-month-old children was carried out in Nonsan, Korea. An interview survey was completed for 71.3% of the children. All data came from a vaccination card or confirmation from a provider.

**RESULTS:**

The hepatitis A vaccination rate was 42.3% for ≥1 dose and 24.7% for 2-dose. The results of the multivariate regression analysis for the hepatitis A vaccination showed that the second (OR=1.6) and third and successive children (OR=3.3) were less often immunized than the first child. Low economic status (OR=1.6), rural area (OR=1.5) and employed mother (OR=1.5) were also correlated with a lower vaccination rate. The hepatitis A vaccination rate was significantly lower in children who had no other vaccinations: measlesmumps-rubella (OR=2.8 for ≥1 dose and 7.3 for 2-dose), varicella (OR=20.2 and 22.0, respectively) and *Haemophilus influenza* type b (OR=14.3 and 13.3, respectively).

**CONCLUSION:**

To prevent outbreaks of clinical hepatitis A by enough herd immunity, a vaccination should be included in the National Immunization Program and a vaccination policy developed and implemented that can overcome the barriers to immunization such as late birth order and a mother's employment.

## INTRODUCTION

Hepatitis A is generally an acute, self-limiting infection of the liver by an enterically transmitted picornavirus, the hepatitis A virus (HAV) [1]. Hepatitis A occurs worldwide, but its prevalence is closely related to hygiene, sanitary conditions and other indicators of a developed country. Although the effective method for controlling enteric infections including hepatitis A is to improve standards of hygiene and sanitation [2], HAV vaccinations have been included in the National Immunization Programs (NIPs) of several developed countries to reduce the number of severe clinical cases in young adults by enough herd immunity [3]. In Korea, hepatitis A did not seem to be a serious disease before 1990 because of the natural immunity derived from hepatitis A infection. Since the mid-1990s, however, the incidence of clinical hepatitis A in young adults has increased. In 2008, 7,895 cases of hepatitis A were detected by sentinel surveillance, which is 3.5 times higher than the number of cases reported in 2007. According to health insurance data, the incidence of HAV was 65.3 cases per 100,000 in 2008, which is 2.3 times higher than the 28.7 cases per 100,000 reported in 2007 (author's study, unpublished data).

Several papers reported this epidemiological shift in the HAV and called for a hepatitis A vaccination in Korea [4, 5]. Since 1997, four inactivated hepatitis A vaccines have been licensed in Korea; Havrix (GlaxoSmithKline Biologicals, Rixensart, Belgium) and VAQTA (Merck & Co., Inc., PA, USA) have been approved for use in the United States, and AVAXIM (Sanofi Pasteur, Lyon, France) and Epaxal (Berna Biotech Ltd., Bern, Switzerland) are licensed in Europe, Canada and several other countries [6]. Havrix and VAQTA are the most used in Korea, but VAQTA has not been used since 2008. In the United States, hepatitis A vaccines were administered to high-risk children in 1996, and in 1999 were included in routine immunization programs in 11 states. They were included in the NIP in 2006 [7]. In Korea, the hepatitis A vaccination is not part of the NIP, and no data exist on the vaccination coverage and the factors related to having a child vaccinated. We report the hepatitis A vaccination rate based on a population-based survey of 12-35-month-old children conducted in 2005 in an area with both urban and rural populations. We also present barriers for hepatitis A immunization and discuss the implications for identifying barriers associated with immunization.

## MATERIALS AND METHODS

### Study population

The population-based survey was carried out between February and April 2005 in Nonsan city. Nonsan has both urban and rural populations, and has a low rate of migration. The target population was 2,188 12-35-month-old children living in Nonsan. The interview survey was completed for 1,561 children (71.3%) and of those 68 did not keep vaccination records; therefore, 1,493 children were included in the analysis. Interviewers were students from medical school or school of public health, who were trained on the study objectives and interviewing methods. We sent a letter to the study subjects before the survey and visited their house. When we could not meet the subjects after three visits, we ascertained the subjects' whereabouts by neighbours or representatives of the town. Many of them did not reside in the town or were living in facilities for orphans or the poor. The primary caregivers of all the children were interviewed and gave written informed consent.

### Vaccination history and analysis

When a vaccination card was available, the interviewer transcribed all immunization doses and dates to a standardized recording form. A report from recall was not accepted without a vaccination card or confirmation from a health provider. During analysis, vaccination rates were split by age group (12-23 months and 24-35 months) and odds ratios (ORs) were adjusted because the vaccination rate can be increased by age. For the economic status, we asked the subjective question "which category is appropriate for your economic status?". When the answers were 'very rich' or 'rich' they were classified as 'high' economic status, 'ordinary' as 'medium' and 'poor' or 'very poor' as 'low'.

For health security, we checked the health insurance or medical aid card and the numbers for confirmation.

Chi-square tests were used to compare rates, and a multiple logistic regression model included all variables that were statistically significant in a univariate analysis except confounding variables. All analyses were conducted using SPSS Ver. 14.0 (SPSS Inc., Chicago, USA).

## RESULTS

The hepatitis A immunization rate for children aged 12-35 months was 42.3% for ≥1 dose and 24.7% for 2-dose. Among children 24-35 months of age, the rates for ≥1 dose and 2-dose were 45% and 35.2%, respectively. The immunization rate was significantly lower in rural areas (47.1% for urban areas and 36.7% for rural areas for ≥1 dose), late birth order (53.0% for the first child, 39.2% for the second child and 24.2 % for the third and successive children for ≥1 dose, and 33.0 %, 21.9% and 11.7% for 2-dose, respectively), large number of siblings (50.5% for no siblings, 44.0% for one sibling and 26.6% for two or more siblings for ≥1 dose, and 28.7%, 27.0 % and 12.9% for 2-dose, respectively) and when the primary caregiver was not the parent (43.6% for parent and 35.2% for others for ≥1 dose, and 25.9% and 18.8% for 2-dose, respectively). No significant difference in the immunization rate was found for gender (Table 1).

The hepatitis A immunization rate was significantly lower in children whose parents had a low economic status for ≥1 dose (p<0.001) and 2-dose (p=0.004). Mothers who were employed and those who unmarried were significantly less likely to have their children vaccinated for ≥1 dose (p=0.002 and p=0.034, respectively; Table 2). The parents' level of health security was borderline significant, and the mother's level of education did not significantly influence whether a child was vaccinated.

The hepatitis A immunization rate was significantly lower in children who had not received other childhood vaccinations. Children who were not immunized against measles-mumpsrubella (MMR) received HAV vaccination 2.8 times less often for ≥1 dose and 7.3 times less often for 2-dose. MMR vaccinees immunized at a public health center were less likely to be immunized against hepatitis A than children immunized at a private clinic (29.8% for a public health centre and 71.0% for a private clinic for ≥1 dose of hepatitis A, and 15.7% and 47.4% for 2-dose, respectively (data not shown).

Children who were not immunized against varicella were also not immunized against the HAV 20.2 times more for ≥1 dose and 22.0 times more for 2-dose. In comparison with the children who had received the *Haemophilus influenza* type b (Hib) vaccination of ≥1 dose, the children who had received no Hib vaccination were at risk of no immunization against hepatitis A (OR=14.3 for ≥1 dose and OR=13.3 for 2-dose Table 3).

The result of the age-adjusted logistic regression model was similar to the multivariate model; the multivariate model included age, birth order, economic status, geographical area and mother's employment status. The analysis revealed that the second child (OR=1.6) and the third or successive child (OR=3.3) were less often immunized than the first child for ≥1 dose of hepatitis A. Low economic status (OR=1.6), rural residence (OR=1.5) and an employed mother (OR=1.5) were significantly decreased for ≥1 dose of the hepatitis A vaccination. The ORs for birth order and economic status for 2-dose were similar to those for ≥1 dose; however, geographical area and mother's employment status were not significant for 2-dose (Table 4).

## DISCUSSION

The immunization rate for hepatitis A among 12-35-monthold Korean children was low: 42.3% for ≥1 dose and 24.7% for 2-dose. Our data indicate that the immunization rate for hepatitis A is significantly lower than that for vaccines part of the NIP, such as MMR (91.2%) and varicella (74.2%, the vaccine was included in the NIP in 2005) [8]. Hib is excluded from the NIP, but the immunization rate for one or more dose was 53.1%, higher than that for hepatitis A.

The hepatitis A vaccine coverage (two doses) in Korea has been estimated to be 41.6% based on the amount of hepatitis A vaccine sold between 1998 and 2006 [9]. However, this study did not account for vaccine doses that were unused and immunization in other age groups, and the figure could be an overestimate.

In 2006, the seroprevalence of the HAV was reported to be 55.6% in children aged 1-4 yr and 47.2% in those aged 5-9 yr [10]. In general, 90-100% of children one year of age and older and adults had protective concentrations of the antibody four weeks after one dose of the vaccination [6]. Anti-HAV has been shown to persist for at least 10-12 yr after vaccination in 5-6-yr-old children [11, 12]. The seropositive rate of hepatitis A was 10.1% (34/337) for 10-24-yr-olds born between 1984 and 1996, i.e., before the hepatitis A vaccine was introduced in Korea, 27.1% for people aged 25-29 yr, 73.2% for 30-39-yr-olds and 96% for 40-49-yr-olds [10]. This means that since the mid-1980s the natural infection rate of hepatitis A has been less than 10% in Korea. Therefore, most Korean children aged 1-9 yr who are immune to hepatitis A received immunity from a vaccination, and natural immunity through a hepatitis A infection is likely to be less than 10%.

In the United States in 2003, vaccination coverage of ≥1 dose was 50.9% for children living in the 11 states in which routine hepatitis A vaccination is recommended, 25% for those living in the six states in which hepatitis A vaccination is considered and 1.4% for the 33 states without a specific recommendation [13]. Israel was the first country worldwide to implement a universal HAV immunization program in which toddlers born after 1997 received two doses of the HAV vaccine at 18 and 24 months [14]. Overall vaccine coverage in the first years after implementation of the program (2001-2002) was ~90% for ≥1 dose and ~85% for 2-dose [14]. In the birth cohort of 2000 in southern Israel, HAV vaccine coverage was 85.5% for ≥1 dose and 74.9% for 2-dose by age three [15]. If the hepatitis A vaccination was recommended as a routine immunization in Korea and free vaccinations were offered at a public health center, vaccination coverage would increase to the level of the NIP vaccines.

In this study, birth order was the factor most highly correlated with hepatitis A vaccination rate. The first child had the highest rate of vaccination followed by the second and then the third and successive children. This finding agrees with other studies on birth order and immunization rates [16, 17]. Birth order might be a proxy parameter of a parent's concern for the child. In this study, economic status was also related to vaccination rate. A hepatitis A vaccination administered in a private clinic is expensive (about $40 for a single dose), and cost is a well-known barrier to immunization [18]. Geographical area was also significantly related to immunization rate: the rate was lower in rural than in urban areas. Because hepatitis A is excluded from the NIP, it is only offered in private clinics, which are more common in urban areas. Furthermore, private clinics are less accessible in rural areas, where the transportation system is less convenient. The barriers for immunization of hepatitis A can be different to other vaccinations in the NIP. In this survey, the significant risk factors for immunization of the NIP were birth order, primary caregiver, mother's employment and marital status; economic status and geographical area were not related to the immunization of the NIP [8]. In the United States, the coverage rate of hepatitis A was higher among children living in urban areas whatever vaccination policy (recommended, considered and not recommended) [13], and in metropolitan areas [19].

Children of employed mothers had a lower vaccination rate, perhaps because those mothers had less time to go to clinics to obtain information about the HAV or to have their children immunized against it. The univariate analysis indicated that the immunization rate was lower when the primary caregiver was not a parent. However, when it was stratified by the mother's employment status, the primary caregiver was no longer significant and mother's employment status was found to be a confounding factor (Table 5). Therefore, the primary caregiver factor was excluded from the multivariate analysis.

The risk factors associated with low immunization coverage can be divided into two groups: parental factors such as poverty, low education and lack of parental care, and provider factors such as health care facility, public health system provision of free vaccines and vaccination policy [18].

This is the first report on the factors associated with the hepatitis A immunization rate in Korea. Our data, however, are derived from one area and thereby are not representative of the entire country. The vaccination histories were based on information taken from vaccination cards. Children without a vaccination record were excluded and many of them were institutionalized; therefore, we might have overestimated the immunization rate.

The WHO suggests that hepatitis A immunization is a cost effective public health tool to control the disease in countries where clinical hepatitis A is a major health problem [20]. The incidence of clinical hepatitis A has recently increased sharply in Korea, and we recommend that the hepatitis A vaccination be included in the NIP. To control the high incidence of hepatitis A in 20-39 yr-olds in Korea, catch-up immunization might also be considered.

In this study, late birth order (less parental concern), low economic status (high cost of vaccination), rural area (low accessibility to a private clinic) and the mother's employment (mother's time commitments) decreased the hepatitis A vaccination rate. The cost and accessibility factors could be alleviated if the hepatitis A vaccination was included in the NIP. However, parental concern and mother's time commitments will continue to affect the hepatitis A immunization rate. Therefore, a vaccination policy that can overcome these barriers should be developed and implemented to increase the immunization rate.

## Figures and Tables

**Table 1:**
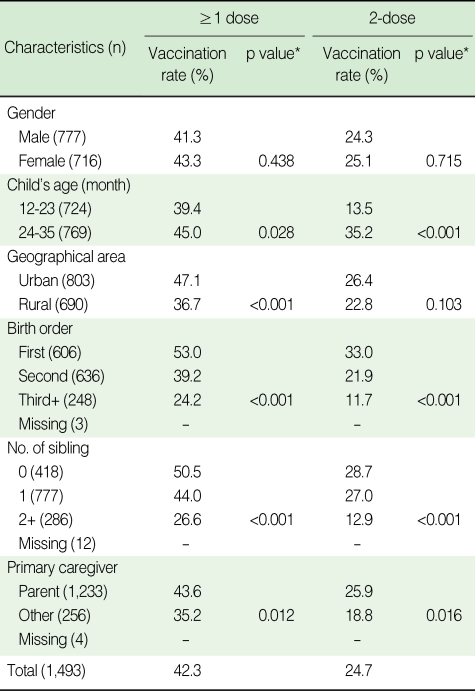
Vaccination rate of hepatitis A by general characteristics of children in Nonsan, 2005

^*^p value obtained by χ^2^ test.

**Table 2:**
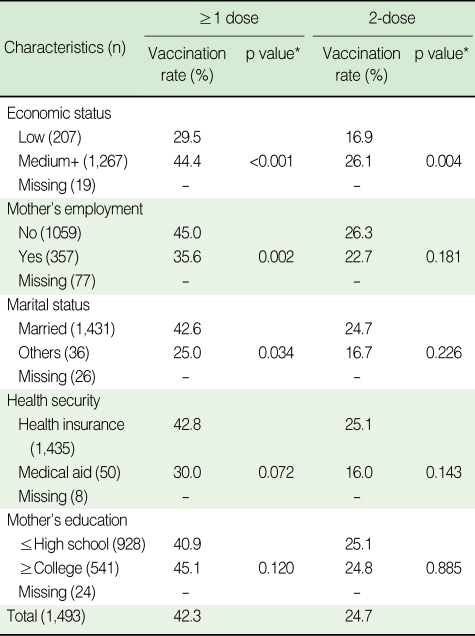
Vaccination rate of hepatitis A by general characteristics of subject's parents in Nonsan, 2005

^*^p value obtained by χ^2^ test.

**Table 3:**
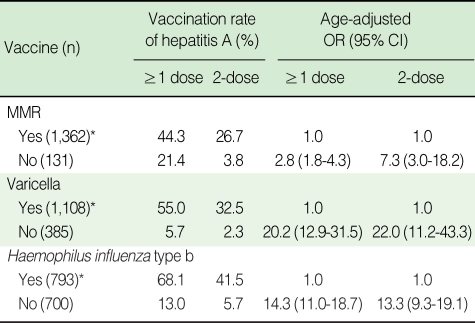
Vaccination rate and risk for non-vaccination of hepatitis A by other vaccinations in Nonsan, 2005

OR, odds ratio; CI, confidence interval. ^*^Children who have vaccination one dose or more.

**Table 4:**
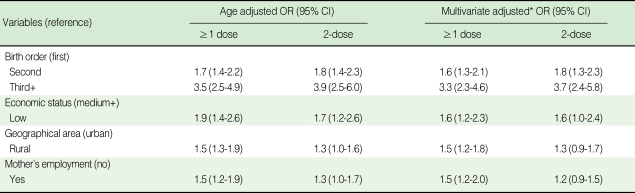
Risk factors for non-vaccination of hepatitis A in Nonsan, 2005

OR, odds ratio; CI, confidence interval. ^*^Logistic regression model included age, birth order, economic status, geographical area and mother's employment.

**Table 5:**
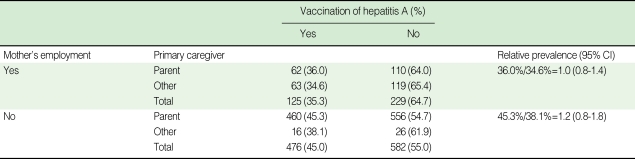
Calculation of relative prevalence after stratifying by mother's employment; observed association of primary caregiver and vaccination of hepatitis A was a result of confounding by a mother's employment

CI, confidence interval.
